# The challenges of urgent radical sigmoid colorectal cancer resection in a COVID-19 patient: A case report

**DOI:** 10.1016/j.ijscr.2020.04.088

**Published:** 2020-05-11

**Authors:** Zhengbin Huang, Jijun Yan, Tian Jin, Xiufang Huang, Guoxiang Zeng, Michael L. Adashek, Xinhai Wang, Jieping Li, Dan Zhou, Zhengqi Wu

**Affiliations:** a Department of General Surgery, Hanchuan People’s Hospital, 1 Renmin Avenue, Hanchuan, Hubei 431600, China; b Department of Pathology, Hanchuan People’s Hospital, 1 Renmin Avenue, Hanchuan, Hubei 431600, China; c Department of Internal Medicine, Sinai Hospital, 2401 W. Belvedere Ave, Baltimore, MD 21215, USA; d Department of Radiology, Hanchuan People’s Hospital, 1 Renmin Avenue, Hanchuan, Hubei 431600, China; e Department of Molecular Biology Laboratory, Hanchuan People’s Hospital, 1 Renmin Avenue, Hanchuan, Hubei 431600, China; f Department of Medicine, Winchester Medical Center, 1840 Amherst Street, Winchester, VA 22601, USA

**Keywords:** COVID-19, Colorectal cancer resection, Surgical risk, Viral transmission, Negative pressure

## Abstract

**Introduction:**

The COVID-19 pandemic presents a unique global health challenge further complicating surgical management of COVID-19 positive patients due to a lack of published literature.

**Case:**

Within we discuss a 48-year-old Chinese man, presenting with acute gastrointestinal obstruction due to sigmoid colonic mass. The patient was screened and tested positive for COVID 19 due to his employment in Wuhan, China at the COVID-19 pandemic epicenter. The patient was subsequently taken for open sigmoid colonic resection, however the case presented multiple challenges due to the patient's COVID-19 positive status.

**Discussion:**

The challenges of surgical management of COVID-19 positive patients exist are four-fold. First the unknown efficacy of pre-surgical risk stratification in COVID-19 positive patients, second the risk of aerosolized COVID-19 transmission during intubation for surgery, third the risk of fecal COVID-19 transmission to surgical staff during large bowel resection, and fourth the post-operative challenges of caring for COVID-19 positive patients.

**Conclusion:**

Further research is needed into these topics, as well as the medical management of COVID-19 surgical patients.

## Highlights

• The first reported case of urgent exploratory colorectal surgery and sigmoid mass resection in a COVID-19 positive patient.
• Discussion of surgical techniques to minimize theoretical viral transmission pre, peri and postoperatively.
• Discussion or risks of fecal transmission of COVID-19 transmission intraoperatively.
• Discussion of personal protective equipment utilization and post-surgical COVID-19 screening among surgical healthcare staff.

## 1 Introduction

Coronavirus Disease 2019 (COVID-19) is a viral respiratory illness caused by the novel coronavirus severe acute respiratory syndrome coronavirus 2. First identified in Wuhan City, Hubei Province, China [1,2], on March 11, 2020, the World Health Organization (WHO) declared COVID-19 a global pandemic [[3], [4], [5]]. Coping with COVID-19 crisis is now a major global challenge effective every aspect of the medical profession.

Multiple other studies have addressed the virology, epidemiology, pathology and critical care management of COVID-19, however surgical management of patients suffering COVID-19 infection remains unclear. In available Pubmed literature review there is a single pre-print article discussing pulmonary surgery in 2 patients with COVID-19 [6]. As of this report there has not been a published peer reviewed literature on the complications and challenges of gastrointestinal surgery in COVID-19 patients. Below we discuss a patient with COVID-19 presenting with acute bowel obstruction due to colorectal cancer, the surgical techniques employed, and the subsequent health of the surgical team. This work has been reported in line with the SCARE criteria [20].

## 2 Case presentation

A 48-year-old Chinese man, with history of positive HBsAg, presented in February 2020 with five days of worsening constipation and lower abdominal pain without fever, cough or subjective dyspnea. Social history was notable for employment in Wuhan, China at the geographic COVID-19 epicenter as a supermarket laborer. Physical exam revealed an age appropriate, ill appearing man, resting in bed without acute cardiopulmonary findings but notable for abdominal distension, periumbilical tenderness and hypoactive bowel sounds. Vital signs were unremarkable with an oxygenation saturation of 95% on room air.

**Fig. 1:**
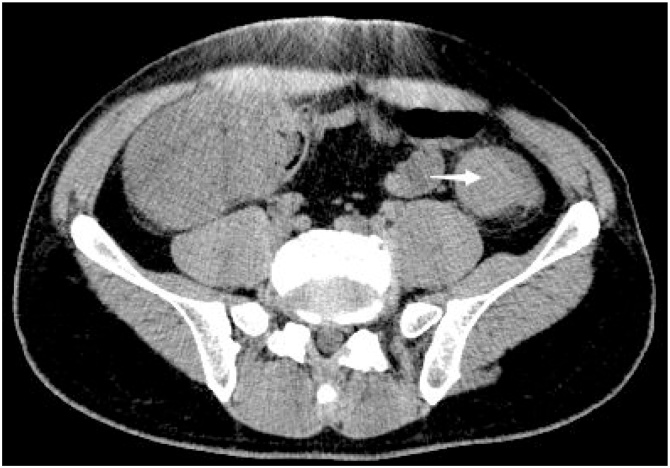
CT of abdomen and pelvis shows obstructive mass in sigmoid colon.

Initial laboratory values demonstrated lymphopenia 0.68 × 10^9^/L (9.6%), elevated Carcinoembryonic antigen 4.32 ng/mL (reference 0–3), and elevated procalcitonin 1.87 ng/mL (reference 0.05–0.5). Computerized tomography (CT) of the abdomen revealed a colonic mass resulting in sigmoid colonic obstruction and large bowel dilatation (Fig. 1).

**Fig. 2:**
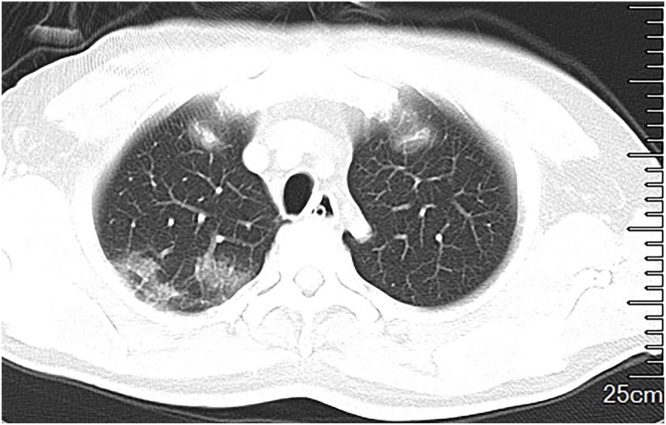
CT of chest shows patchy ground glass opacities in right upper lung, peripheral field.

The patient’s social history necessitated COVID-19 evaluation, particularly with elevated procalcitonin, lymphopenia and ground glass opacities in the right upper lung on chest CT (Fig. 2). COVID-19 testing was not available prior to surgery and performed afterward.

Risks and benefits of surgery for acute sigmoid colonic obstruction in the circumstance of COVID-19 were discussed, and the surgery proceeded in a negative pressure operating room. The team followed proper protocol for personal protective equipment for both airborne and contact precautions. First the team performed hand hygiene prior to donning shoe cover, cap, N95 mask, goggles and isolation gown. In the second step, the gloves were sanitized, surgical gown donned and second set of sterile gloves placed. The patient was induced under general anesthesia and intubated orally. Radical open sigmoidostomy and colonic decompression superior to the obstructing mass was performed, after which a stapler was utilized to perform primary anastomosis and defunctioning ileostomy was created.

**Fig. 3:**
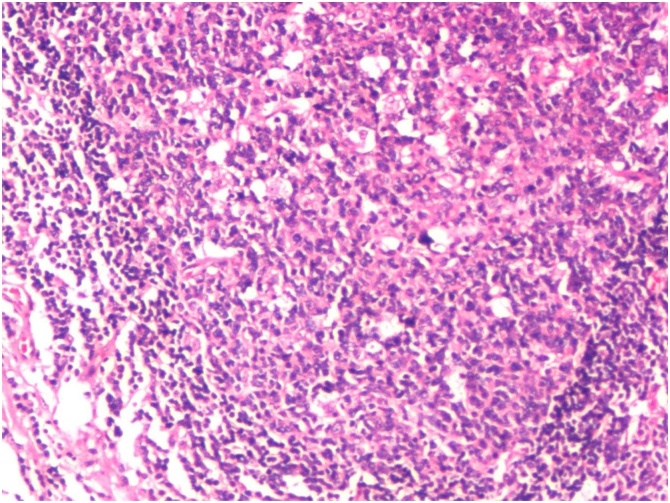
Hematoxylin-eosin staining of colon tumor shows moderately differentiated malignant cells invading the seroma.

Throughout the case, negative pressure in the operating suite was maintained below −5 Pa [7]. To minimize potential viral transmission the patient was extubated and recovered in the operating suite prior to arriving in the post-anesthesia care unit which was not equipped with negative pressure. All surgical tissue and waste were properly labeled with COVID-19 labeling and double sealed. Surgical pathology revealed adenocarcinoma T4aN0M0 (Fig. 3).

COVID-19 pharyngeal swab testing was obtained and PCR positive for COVID-19. Throughout his clinical course the patient improved, with post-operative (post-op) chest CT demonstrating resolution of ground glass opacities. Subsequent COVID-19 PCR testing on post-op days 15 and 16 were negative. The patient was discharged from the hospital on post-op day 18 without further complications and scheduled for outpatient follow up.

Given concerns regarding potential transmission, the surgical staff were monitored for symptoms such as cough, fever, chills, myalgia or diarrhea 2 weeks post operatively without evident stigmata of COVID-19 infection. On post-op day 7 team members underwent screening chest CT imaging for further screen for COVID-19 without any noted imaging abnormalities.

## 3 Discussion

COVID-19 presents in a myriad of severities. Wu and McGoogan et al. reported among 72,314 COVID-19 cases, 81% were mild (defined as absent or mild pneumonia), 14% severe (hypoxia, dyspnea, >50% lung involvement within 24–48 hours), 5% critical (shock, respiratory failure, multiorgan dysfunction), and 2.3% fatal [8]. Given the increasing incidence of COVID-19 in the general population, the acute surgical population too will experience a surge in the number of patients with active COVID-19 infection. Current screening guidelines recommend discussing fever, chills, cough, known COVID-19 positive contacts, and travel history to increase pre-test probability of a patient’s likelihood to test positive for COVID-19. However, pre-surgical risk stratification for COVID-19 screening management has not yet been well defined.

Pre, peri, and post-surgical procedure to minimize viral transmission to healthcare staff, as well as anticipation of a patient’s potential for pulmonary complications are among the most critical concerns regarding these patients. It is current consensus that COVID-19 is transmissible through aerosolized droplets and physical contact, although airborne transmission remains a concern.

The rising incidence of COVID-19 will likely place a previously unforeseen strain on infection prevention in large community centers where personal protective equipment (PPE) shortages have been reported worldwide. Leung et al. demonstrated the efficacy of surgical masks to reduce coronavirus detection and viral copies in large respiratory droplets as well as aerosols, suggesting surgical face masks may limit viral transmission of COVID-19 suspected patients in transport [9]. Additionally, studies have shown tracheal intubation can increase risk of viral transmission and suggested rapid sequence intubation may reduce transmission risk [10,11].

Intraoperatively, enhanced PPE (especially N95 mask and goggles) for surgical staff have been recommended by the Chinese Center for Disease Control. Patient risk categorization, reconstruction of operating room (preferably to negative pressure −4.7 Pa in the main room and −1.2 Pa in the anteroom), reorganization operating room workflow process are all likely to become key elements for the containment of viral transmission [7,12].

**Fig. 4:**
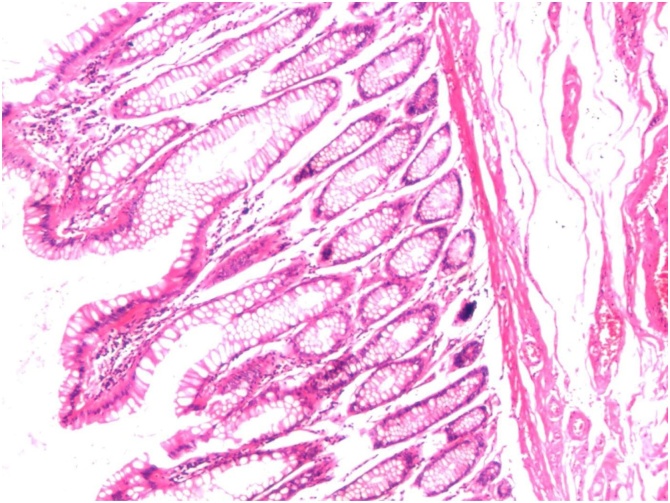
Hematoxylin-eosin staining of adjacent descending colon shows scattered lymphocytes and plasma cell infiltration.

In colorectal cancer, laparoscopy-assisted radical surgery has typically been the surgical modality of choice [13] with the understanding that conversion to an open surgical approach may be necessary to address difficult cases. Due to anticipated complexity and potential for bowel perforation, this case was addressed initially with open surgical technique. COVID-19 viral RNA has been detected in the stool of a patient in the USA [14] and it is possible COVID-19 could be transmitted via fecal–oral route, through the angiotensin-converting enzyme 2 (ACE2) cell receptor [15]. Although our patient was COVID-19 positive, adjacent bowel tissue of our patient appeared histologically unaffected with the exception of scattered lymphocyte and plasma cell infiltration (Fig. 4). However, strict precautions such as minimal bowel exposure, bowel decompression and proper fecal disinfection were taken during the surgery to prevent viral dissemination to surgical staff. The patient’s stool was not tested for COVID-19 due to critical shortage of testing reagents.

Postoperatively, it has been difficult to anticipate the outcomes of patients infected with COVID-19 due to lack of published data. Recently two patients with active COVID-19 infection underwent thoracoscopically lobectomy, and though stable prior to surgical intervention noted 50 % post-operative mortality [6]. Further studies are required, and the small sample size of this case series anecdotal, although worrying at this time. Thankfully for our patient, his COVID-19 remained mild post-surgery.

Unfortunately, no specific antiviral agents in the treatment of COVID-19 have definitely been proven effective at the time of this paper. Remdesivir may have the greatest potential of inhibiting COVID-19, but its efficacy and safety require further evaluation [16]. In China, several clinical trials of chloroquine or hydroxychloroquine have demonstrated promising results in inhibiting exacerbation of pnumonia, promoting viral clearance, and shortening disease course [17,18]. Hydroxychloroquine-azithromycin combination therapy trials have also been implemented although finalized data is pending [19].

Finally, testing of clinical staff involved in patient care is again a difficult issue to address. Ideally, testing of all staff involved in patient care would allow for optimal minimization of viral transmission. However, lack of testing reagents and test kits worldwide limit this approach. Alternatively, due to availability screening chest CT was utilized among surgical staff per CCDC recommendations at that time. American College of Radiology guidelines as of March 11, 2020 have recommended against this approach due to radiologic exposure and lack of sensitivity. Further studies are needed to identify if the guidelines regarding COVID-19 screening are applicable to surgical staff in high risk aerosolizing environments such as operating suites.

## 4 Conclusion

In conclusion, COVID-19 remains a global health challenge. The efficacy of perioperative risk stratification in COVID-19 positive patients with varying degrees of COVID-19 infection remain unknown and requires further investigation. Although fecal COVID-19 RNA has been detected, surgical pathology did not note COVID-19 in adjacent bowel tissue, but this finding has unclear clinical implications at this time. Regardless, all surgical tissue and surgical waste removed in both large and small bowel surgeries will likely have fecal contamination and should be treated as a potential infectious source of COVID-19 and disposed of accordingly. Finally, further investigation is needed into post-operative management of COVID-19 patients, medical management of COVID-19 and appropriate screening for surgical staff involved in operating on COVID-19 patients.

## Declaration of Competing Interest

All authors declare no conflicts of interest.

## Sources of funding

No funding involved in this case study.

## Ethical approval

This is a case report. It does not require Ethical approval. The patient consents for the publication.

## Consent

Written informed consent was obtained from the patient for publication of this case report and accompanying images. A copy of the written consent is available for review by the Editor-in-Chief of this journal on request.

## Authors contribution

Zhengbin Huang: Study concept, data collection, data analysis and interpretation.

Jijun Yan: Data collection, data interpretation.

Tian Jin: Data collection, data interpretation.

Xiufang Huang: Data collection, data interpretation.

Guoxiang Zeng: Data collection, data interpretation.

Michael L. Adashek. Critical review and editing the article.

Xinhai Wang: Data collection, data interpretation.

Jieping Li: Data collection, data interpretation.

Dan Zhou: Data collection, data interpretation.

Zhengqi Wu: Writing the article, final approval of submission.

## Registration of research studies

Name of the registry: **Research Registry**

Unique identifying number or registration ID: researchregistry5529

Hyperlink to your specific registration (must be publicly accessible and will be checked): https://www.researchregistry.com/browse-the-registry#home/registrationdetails/5ea0a8abef05070015be1856/

## Guarantor

Zhengbin Huang.

## Provenance and peer review

Not commissioned, externally peer-reviewed.
